# Supplementary material for: Genetic Variants Associated with Drug Resistance of Cytomegalovirus in Hematopoietic Cell Transplantation Recipients
Source: Viruses. 2023 May 30;15(6):1286. doi: 10.3390/v15061286 (PMC10305717; doi:10.3390/v15061286)
Supplement: Supplementary file 1 [file viruses-15-01286-s001.zip › viruses-2401615-supplementary.pdf]

Table S1. Patients with resistance variants in CMV *UL97* and *UL54* genes

	**Patient #1**	**Patient #2**	**Patient #3**	**Patient #4**	**Patient #5**
Gender, age (years)	F/50	M/28	F/13	M/53	F/17
Primary disease	MDS	ALL	ALL	AML	MDS
HCT source	Peripheral blood	Cord blood	Peripheral blood	Cord blood	Peripheral blood
Donor type	Unrelated	Unrelated	Unrelated	Unrelated	Related
HLA-matching	Matched	Matched	Matched	Matched	Mismatched
Letermovir prophylaxis	No	No	No	Yes	No
CMV D/R serostatus	D?/R+	D?/R+	D-/R+	D?/R+	D+/R+
Initial antiviral regimen	Valganciclovir	Ganciclovir	Ganciclovir	Ganciclovir	Ganciclovir
Period of development of refractory CMV DNAemia (days)	65	68	44	124	40
Resistant variant in the *UL97* gene	A594V	M460I, A594V	L595F	C603W	L595W
Resistant variant in the *UL54* gene	A809V	No	No	No	No
Modification of initial antiviral regimen	Yes	Yes	No	Yes	No
Secondary antiviral regimen	Foscarnet	Foscarnet	.	Foscarnet	.
CMV clearance (days)	Yes (239)	Yes (256)	Yes (168)	Yes (142)	Yes (133)
Acute Graft vs. Host Disease	Moderate	Moderate	Severe	Severe	Moderate
CMV organ disease	Colitis	Colitis	Retinitis	Colitis<br>Retinitis	Retinitis
Peak CMV viral load (IU/ml)	3,135,692	36,080	310,464	64,569	467,696
Outcome	Alive	Dead	Alive	Dead	Dead

Table S1. Continued

	**Patient #6**	**Patient #7**	**Patient #8**	**Patient #9**	**Patient #10**
Gender, age (years)	M/23	F/64	M/59	F/67	F/5
Primary disease	NHL	ALL	AML	AML	HLH
HCT source	Peripheral blood	Peripheral blood	Peripheral blood	Peripheral blood	Peripheral blood
Donor type	Related	Unrelated	Related	Related	Unrelated
HLA-matching	Mismatched	Matched	Mismatched	Mismatched	Matched
Letermovir prophylaxis	No	No	No	No	No
CMV D/R serostatus	D+/R+	D+/R+	D-/R+	D-/R+	D?/R+
Initial antiviral regimen	Ganciclovir	Ganciclovir	Ganciclovir	Ganciclovir	Ganciclovir
Period of development of refractory CMV DNAemia (days)	57	51	189	266	70
Resistant variant in the *UL97* gene	No	No	L595W	A594V	L595W
Resistant variant in the *UL54* gene	A809V	F412L	F412L, V787L	No	No
Modification of initial antiviral regimen	Yes	No	Yes	Yes	Yes
Secondary antiviral regimen	Foscarnet	.	Foscarnet	Foscarnet	Foscarnet
CMV clearance (days)	No	No	No	Yes (155)	Yes (125)
Acute Graft vs. Host Disease	Severe	Severe	Severe	Moderate	No
CMV organ disease	Colitis<br>Retinitis<br>Encephalitis	No	gastritis<br>colitis<br>encephalitis<br>pneumonitis	gastritis<br>colitis<br>retinits	No
Peak CMV viral load (IU/ml)	865,252	386,720	14,690,000	806,000	1,079,000
Outcome	Dead	Dead	Dead	Alive	Alive

MDS, myelodysplastic syndrome; ALL, acute lymphoblastic leukemia; AML, acute myeloid leukemia; NHL, non-Hodgkin lymphoma; HLH, Hemophagocytic lymphohistiocytosis; HCT, hematopoietic cell transplantation; HLA, human leukocyte antigen; CMV, cytomegalovirus; D+, donor seropositive; D-, donor seronegative; D?, donor serostatus unknown; R+, recipient seropositive.
